# Effects of Deep Brain Stimulation on the Lived Experience of Obsessive-Compulsive Disorder Patients: In-Depth Interviews with 18 Patients

**DOI:** 10.1371/journal.pone.0135524

**Published:** 2015-08-27

**Authors:** Sanneke de Haan, Erik Rietveld, Martin Stokhof, Damiaan Denys

**Affiliations:** 1 Academic Medical Center, Department of Psychiatry, University of Amsterdam, Amsterdam, the Netherlands; 2 Amsterdam Brain and Cognition, University of Amsterdam, Amsterdam, the Netherlands; 3 Institute for Logic, Language and Computation, Department of Philosophy, University of Amsterdam, Amsterdam, the Netherlands; 4 The Netherlands Institute for Neuroscience, Royal Netherlands Academy of Arts and Sciences, Amsterdam, the Netherlands; Mayo Clinic, UNITED STATES

## Abstract

Deep Brain Stimulation (DBS) is a relatively new, experimental treatment for patients suffering from treatment-refractory Obsessive Compulsive Disorder (OCD). The effects of treatment are typically assessed with psychopathological scales that measure the amount of symptoms. However, clinical experience indicates that the effects of DBS are not limited to symptoms only: patients for instance report changes in perception, feeling stronger and more confident, and doing things unreflectively. Our aim is to get a better overview of the whole variety of changes that OCD patients experience during DBS treatment. For that purpose we conducted in-depth, semi-structured interviews with 18 OCD patients. In this paper, we present the results from this qualitative study. We list the changes grouped in four domains: with regard to (a) person, (b) (social) world, (c) characteristics of person-world interactions, and (d) existential stance. We subsequently provide an interpretation of these results. In particular, we suggest that many of these changes can be seen as different expressions of the same process; namely that the experience of anxiety and tension gives way to an increased basic trust and increased reliance on one’s abilities. We then discuss the clinical implications of our findings, especially with regard to properly informing patients of what they can expect from treatment, the usefulness of including CBT in treatment, and the limitations of current measures of treatment success. We end by making several concrete suggestions for further research.

## Background

### I.A. Introduction

Deep Brain Stimulation (DBS) is a relatively new, experimental treatment for patients suffering from treatment-refractory Obsessive Compulsive Disorder (OCD). The effects of treatment are typically assessed with psychopathological scales that measure the amount of symptoms, such as the Yale-Brown Obsessive-Compulsive Scale (Y-BOCS) [1], the Hamilton Anxiety Rating Scale (HARS) [2], and the Hamilton Depression Rating Scale (HDRS) [3]. Global functioning is often assessed by means of the Global Assessment of Functioning (GAF) [4]. These are quite a few scales already, but still clinical experience learns that these scales do not yet capture all the relevant changes that patients experience during DBS treatment. First of all, the effects of DBS are not limited to OCD symptoms only: patients for instance report changes in perception, feeling stronger and more confident, and doing things unthinkingly. These changes fall outside of the scope of not only the Y-BOCS, but also of the anxiety and depression scales. Besides, scales and experiences are sometimes incongruent: some patients in our study whose scores on the Y-BOCS scales had hardly changed, reported that their lives had become much more liveable, to the extent of making the difference between being suicidal or not. Others did show improvements on the Y-BOCS scale, but still reported considerable suffering and feeling highly disabled.

The aim of our study is to get a better overview of the variety of changes that OCD patients experience during DBS treatment. What are their experiences of the effects of DBS, not just with regard to their symptoms, but rather in general? For that purpose we conducted a qualitative study in the form of in-depth, semi-structured interviews with 18 OCD patients who are being treated with DBS. These interviews have the advantage that participants are not restricted to pre-given categories and types of answers, as in the standardized scales mentioned above.

The setup of this article is as follows: we first give a short introduction to OCD and DBS as well as a short overview of what is known so far about psychosocial effects of DBS with OCD patients. In the second part, we explain the methodology of our study. In the third part, we present the results. We have grouped the experiences of the participants into four main domains, namely changes with regard to (a) self-experiences of the person, (b) their experience of their surroundings, including social interactions, (c) the way in which participants interact with their surroundings, and (d) the existential stance: the evaluative relation of participants to themselves and their situation. Each of these domains consists of several sub-themes, which we illustrate with quotes from the participants. In section four, we discuss these results. We highlight what is new in our findings, and we propose an interpretation of the coherence between the changes. In section five, we turn to the clinical implications of our study. We offer some suggestions for optimizing treatment procedures, and we argue that our findings indicate the need for a debate on what should be the criteria to consider patients to be ‘responders’ or ‘non-responders’. We end our article by making several suggestions for future research.

### I.B. Obsessive-Compulsive Disorder

People with OCD suffer from either obsessions, or compulsions, or both. Obsessions are recurrent, intrusive thoughts or images that are unwanted, such as sexual or aggressive visions. They cause anxiety and distress, and people try to ignore or suppress them, or to neutralize them through some other thought or action [5]. Such neutralizing thoughts or actions are compulsions: repetitive (mental) acts, such as hand washing, ordering, checking, counting, or praying, that people feel driven to perform in order to prevent or reduce anxiety or distress or to prevent some dreaded event or situation, even though it is clear that these acts are either unrealistic or excessive [5]. Moreover, a diagnosis of OCD requires that people spend more than one hour a day on their obsessions or compulsions, or that they cause marked distress, or lead to impairments in functioning. Furthermore, the diagnosis requires a specification of the extent to which patients have insight in the unrealistic or disproportional character of their obsessions and compulsions.

With regard to the phenomenology of OCD, several characteristics stand out. First of all, the urge of *having to* perform these compulsions is very strong. As one of our participants explained it: ‘if you’re swimming, and somebody pushes your head under water, than everything in your body screams: “I have to get up to get air”. You HAVE TO—otherwise you will choke. I find that is comparable to compulsions.’ (P2). It does not matter that patients rationally know it is nonsense and also find it ridiculous themselves: the anxiety overrules this knowledge. What they know does not affect how they feel. As one participant remarked: ‘someone who is depressed also won’t be cured by telling him a joke. That doesn’t work. (…) [In OCD] Realistic arguments don’t work for you anymore.’ (P4). In fact, their insight into the senselessness of their compulsions adds to their suffering: ‘It is repulsive. You don’t want it, and you have to do it. You’re actually doing something against your own will.’ (P6).

Another characteristic of these compulsions is that patients need to go on with them ‘until it feels right’. That is, until the tension or anxiety have tempered or patients feel the action is ‘completed’ [6,7]. Moreover, compulsions need to be performed with uttermost attention and focus: these are not mindless automatisms, but highly controlled rituals [8]. In case patients get distracted, they even need to start all over again.

Usually, the compulsive rituals have started off as relatively harmless means to feel calm and in control. But both the obsessions and compulsions can get out of hand, and spread like wildfire through someone’s life. OCD can become very severe: patients may spend all of their waking hours cleaning, or checking, or ordering. They may avoid going outside of the house at all, out of fear of germs. They may wash their hands until their skin peels off. And perhaps worst of all, the OCD is never ‘off’: the fears, the thoughts, the tension; they are constantly present, with everything the patients do. They can never escape from it. Participants explain that they feel trapped inside their own head.

In fact, the World Health Organisation lists OCD as one of the twenty most disabling diseases [9]. OCD affects approximately 2% of the general population [10]. About 50 to 60 percent of patients respond well to treatment with Cognitive Behavioural Therapy (CBT) and/or medication [9,11]. About 10 percent of the patients however, do not respond to any of these treatments [11]. For these patients, Deep Brain Stimulation may be a treatment option.

### I.C. Deep brain stimulation for OCD

Deep brain stimulation involves the implantation of electrodes in the brain which give a continuous electrical pulse to modulate specific brain areas. The activity of the electrodes can be programmed externally with a portable appliance communicating with the pulse generator through telemetry. The programming facility has the advantage that, after implantation, the stimulation can be optimized in order to increase the therapeutic effect and to decrease side effects. However, a limitation is that finding optimal stimulation settings can be a lengthy procedure, which may require several months, since the optimal settings in terms of contact points and voltage (and possibly bandwidth) differ per patient.

DBS started as treatment for movement disorders, mainly for Parkinson’s Disease. In the last decade, the use of DBS for treatment resistant psychiatric disorders such as Major Depression, Tourette Syndrome, and OCD is being tested [12–17]. Since DBS is still a new and experimental form of treatment for psychiatric disorders, there is no consensus yet on which brain region would be the best target for the various disorders [18]. (For more on the neurobiology and mechanisms of action of DBS, see: [19–23]) The average overall responder rate for patients with treatment resistant OCD is around 50% (de Koning, Figee et al. 2011). Being a responder is defined as having a reduction of 35% or more compared to one’s pre-operative score on the Yale-Brown Obsessive-Compulsive Scale (Y-BOCS) [1]. At the Academic Medical Center (AMC) in Amsterdam 42 OCD patients are currently being treated. All patients are stimulated in the nucleus accumbens. An earlier study has shown that this treatment is effective in approximately 57% of the OCD patients [11].

### I.D. Known effects of DBS on experiences of OCD patients

There have been several studies into the effectiveness and impact of DBS for OCD patients. Here we sum up the main findings with regard to the patients’ experiences. First of all, it was found that DBS leads to a sustained diminishment of anxiety (measured by the HARS) and an improved mood (measured by the HDRS) with stimulation in the anterior limb of the internal capsule (AL/IC) [24], the nucleus accumbens (Nac) [11], and ventral capsule/ventral striatum (VC/VS) [14]. Some studies found a transient increase of anxiety with stimulation in the VC/VS [14], and of panic and fear with stimulation in the AL/IC [25]. Many studies report that some patients experienced a transient state of hypomania, usually lasting no more than a few days, with stimulation in the VC/VS [14,26], in the VC [27], in the subthalamic nucleus (SN) [28], in the internal capsule (IC) and Nac region [29], or in the Nac [11,30]. In one case, a patient experienced a full-blown mania after stimulation of the AL/IC and the Nac [31]. Both mania and hypomania disappeared after adjusting the DBS settings. Also, experiences of hyper-sexuality following AL stimulation have been reported [32], and an increase in libido with stimulation in the Nac [11], as well as increased impulsivity with stimulation in the Nac [33].

With regard to effects of DBS on cognition, most studies report that there are no significant effects of stimulation in the VC/VS [14,26,34] and in the AL/IC [24]–with the cautionary remark that all sample sizes are small. Denys and colleagues [11], however, found mild forgetfulness and word finding problems in several patients who were stimulated in the Nac. Other findings concern case reports on the effects of DBS of the Nac on remission of alcohol dependency [35] and smoking cessation [36,37], as well as weight loss [37]. Another case report recounts the change of musical preference of a patient following DBS in the Nac [38].

A few studies have looked into the global functioning and quality of life of OCD patients following DBS treatment, in the VC/VS [26], and in the Nac [30,39]. The study of Ooms and colleagues [39] found an improvement in three out of four domains on the brief version of the WHO Quality of Life Scale [40]: the physical, psychological, and environmental (finances, housing, opportunities for recreation, etc.) domains significantly improved both after eight months and three to five years of stimulation of the Nac. There was, however, no significant improvement of the social domain (which is comprised of items on social support, sexual life, and personal relationships). Greenberg and colleagues [14] did find positive changes in social domain, as measured by the GAF [4]. Interestingly, Ooms and colleagues [39] found that the quality of life on the first three domains improved for *both* responders and non-responders. This finding raises questions about the proper construction of or criteria for being a (non-) responder. We will come back to this issue in Section V.

### I.E. What is missing in the literature?

Even though OCD patients undergoing DBS treatment are very well investigated, what is missing in the literature so far is a broad investigation of all changes in experiences–not only those that are measured by standard psychopathological and functional scales. Several case reports provide an insight into some of these changes that fall out of the scope of the scales. But these are only anecdotal investigations. Moreover, no research has been done that focussed on the patients’ own perspective on their treatment. Given that DBS is only a recent and experimental form of treatment for OCD, which has only been used for a small number of patients worldwide, much of the effects of DBS are still unknown. In order to systematically explore and describe the changes in the experiences and life-world of OCD patients following DBS treatment, we have set up a qualitative study; conducting semi-structured in-depth interviews with 18 participants. Before reporting the results, we first describe the study design and methods.

## Study Design & Methods

### II.A. Aim

The aim of this study was to explore and describe the changes in the experiences and life-world of OCD patients following DBS treatment.

### II.B. Methodology

The participants were interviewed by the first author, using in-depth, semi-structured qualitative interview methods. Apart from the interviews, the first author was also immersed at the psychiatric department of the AMC for the past three years and was present at patients’ meetings, therapists’ meetings, and several therapy sessions. The first author is a philosopher with training and experience in conducting qualitative semi-structured interviews. She was involved with the participants only as a researcher, not as a therapist.

This study roughly follows the ‘grounded theory approach’ [41]. That is; the data collection is primary and we developed our theoretical concepts by making sense of these data, in a continuous back and forth between data collection and analysis. Our background in phenomenology and ecological psychology, however, inevitably shaped our analysis, and the kinds of concepts that we use when interpreting the results (see section IV).

### II.C. Participants and setting

Eighteen people participated in the study; 10 women and 8 men, ranging in age from 26 to 65 years. Since some participants have almost completely recovered from OCD, we use the general term ‘participants’ rather than ‘patients’. The interviews took place 6 to 91 months after the DBS operation. Eleven participants were responders at the time of the interview. All participants met the inclusion criteria for DBS treatment at our hospital. For the full list of inclusion and exclusion criteria see: [11]. The interviews took place at the psychiatric hospital, except for two participants (P7, P14) who were interviewed at least once at their own home. Participants were generally interviewed alone, in two cases (P5, P17) the participant’s partner was present, but did not take part in the interview. Participants were recruited personally by the first author, after they gave their permission to contact them via their therapists at the AMC. They received oral and written information, and confirmed their participation by signing an informed consent. They could at all times withdraw from the study. The study was approved by the medical ethics committee of the AMC (approval number: W11_113 # 11.17.1006).

We applied ‘purposeful sampling’, which means that we selected a diverse as possible group of participants to in order to get a broad overview of the various kinds of changes that people experienced. Sometimes therapists would recommend including specific patients because they had special experiences and/or were particularly good at expressing themselves. See S1 Table–Overview of Participants for a more elaborate overview of the participants.

### II.D. Data collection

Firstly, all participants were interviewed at least once, using a semi-structured topic list (see S1 Text—Topiclist). The interviews were videotaped (in one case audiotaped (P15)) and transcribed verbatim. The interviews took one to three hours, with a mean duration of one and a half hour. The general themes of questions were chosen as reflecting basic aspects of people’s lives and life-world. In line with qualitative interview methods, the interviews started off with general open questions, subsequently exploring the answers using mainly open questions, with only incidental use of probing questions and examples.

### II.E. Data analysis

The first author analysed the interviews through open coding; e.g. labelling fragments using mostly the participants’ own words (‘in vivo codes’), or sometimes referring to a theoretical concept (‘constructed codes’). MAXQDA 10 software was used for the analysis. To diminish personal bias, three interviews (P3, P4, and P14) were coded together with the second author. All authors had regular discussions amongst each other and with affiliated researchers about the interpretation of the results. Furthermore, all participants were sent an earlier version of this article so that they could provide comments, amendments and corrections. They could do so via email, telephone, or at one of the meetings we organized for that purpose. The present text is adjusted according to their feedback.

## Results

### III.A. Overview of themes

In this part, we will give an overview of several themes that emerged from the interviews, illustrated by quotes. We have divided the reported changes into four groups: changes with regard to (1) person; (2) life world & social interactions; (3) characteristics of the person-world interaction; and (4) existential stance. We have chosen these four domains, because each of them highlights another aspect of the overall changed way of being in the world of the participants: as persons change (1), so does their experience of the (social) world (2), and the specific way in which they interact with their (social) environment (3): that is, how flexible, automatic etc. their interactions go. The existential stance (4) refers to the evaluative relation of participants to themselves and their situation: to their attitude towards the changes, or towards the DBS device for instance. For a more elaborate discussion of the philosophical background of these four domains, see: [42].

Some preliminary remarks: First, note that the changed experiences listed here are not restricted to obsessive or compulsive behaviour only, but rather concern the person and her experience of the world *in general*. And secondly, there are of course many connections between various of these experiences: being more daring (1g) is for instance likely to be related to the experience of increased self-confidence (1h), as well as to doing things unthinkingly (3b). Moreover, changes in one domain may lead to changes in other domains as well. For instance, when participants spend less time on their compulsions, this in itself may have an impact on their social life. We will come back to the connections and influences between the changes in the Discussion.

#### (1) Person

(a) Less anxious. In line with what is known from previous studies (cf. section I.D), the most common experience after appropriate (e.g. most beneficial for this particular patient) settings of the DBS have been established is that of being *less anxious*. This is often felt immediately, in the next seconds or minutes, or else in the course of a few hours. The general level of anxiety drops, and many compulsion-related situations that used to be very frightening are now less so.

‘I felt very emotional [after the DBS had been switched on], and I felt I was becoming happier. Yes, and clearly less anxious.’ (P2)

‘Yes, that is really the central theme; that I started to worry less about things that I used to worry about for days… and really avoiding things, but that has.. And I find now, now I have this [the DBS] for more than a year, that it continues to become better. (…) No, like I said, it has not completely disappeared, but I don’t really get anxious about it anymore. And the thought can be there, that I would normally go on to perform a compulsion to wash my hands, but I just don’t do it anymore. It comes up, and it fades out, that thought. So.. I am not yet that I can say ‘I have none of these thoughts anymore’. I don’t have that. I don’t know whether that will ever go away, but at it is now, it is doable, so to say. Really.’ (P17)

(b) Improved mood & hopeful. For many participants, the lessening of anxiety goes together with an improvement of their mood: they feel more cheerful, happy, and are better able to enjoy things. Several participants explain that they feel lighter, as if a burden has fallen off:

‘And in November or so, then I noticed something… And what it was exactly.. As if.. it became lighter… I always have a heavy backpack, at my head actually, at my head I felt a brick–as if that became lighter. I had more time to do other things. Not a whole lot of time, small, small things.’ (P8)

Participants (8) also commonly note that they feel more hopeful, and optimistic, and that they now see perspectives, which they did not see before.

‘I have started to look at the possibilities to start a little online store (…) So then you see all these possibilities, and I have also started to look into things. And then I think: oh I can do this, I can build that, and that’s actually not so hard… If it [DBS] had not been, then I maybe would not have even thought of doing something like that, or maybe I would have thought about it, but then like: that will never work out anyway. But now I think: oh, I can give it try, and if it doesn’t work out, that’s okay, but if it does work out, that would be nice.’ (P3)

(c) More easy & careless. Another common experience is that many participants become more easy, and loose, and also indifferent and careless. This is not limited to their symptoms, but rather constitutes a general change that concerns all of their interactions. Participants note that they have become better at letting things go, and at relativizing. Participants worry less about things that they used to worry about, and stop avoiding compulsion-related situations:

‘For example, I used to be terrified of coffee, everything that had to do with coffee; then I went away. So all the places where coffee is served, I wouldn’t go there. And that’s almost everywhere. And now, if coffee gets spilled on my clothes, or on my hand, than I just swipe it off, and I don’t get into panic, I feel like: well, so what.’ (P10)

Several participants report that when they do get into a difficult situation, they find they can handle it better than before; it’s still difficult, but they don’t panic. Two participants have become less perfectionist, and one of them has even become sloppy, for instance with construction work around the house. Becoming more indifferent also applied to social situations: several participants noted that they cared less about the troubles of other people, and found they had less patience to listen to nagging.

(d) Self-confidence & self-reliance & trust. Several participants (7) report that they are more self-confident in social interactions. Besides, in a more basic sense they trust themselves more: they rely more on their own senses, judgments, and capacities. That is, they trust in what they see, and in their own actions: for instance that they really did not spill anything, that the door they have locked is really locked.

‘Of course, every evening, I still make my tour of inspection through the house, before I go to bed: I check the oven, the coffee-machine, that kind of things. But I am not looking ten times anymore; off is off.’ (P17)

‘A lot of things are just right, I can *see* that now… I think it just comes across or something.’ (P8)

Participants also trust in their own judgments more: having less doubts, being more decisive and relying on what they think rather than depending on the judgment of others. With regard to their capacities too, they trust themselves that they can do things, that they can handle situations that they used to avoid.

(e) More daring & strong. A large majority of 13 participants mentioned that they dared to plan and to do things again. They used to avoid making plans, because it usually did not work out anyway because of the compulsions. Now, related to the hope and optimism, participants started to make plans, either for the next days, or even for the next years. And they also in fact dared to do more things, were less avoidant. One participant for instance had always regretted that he had not been able to go to university because of his severe OCD, and now, twenty years later, he arranged to go to university after all:

‘Now I can see myself walking around there, and attending lectures, and asking a question–because now I would dare to ask the lecturer. Yes, now is the time, yes. Because back then, I never dared these things…’ (P13)

Several of these participants also reported that they felt stronger, and had more fighting spirit:

‘Apart from love, the DBS certainly has done something, because I am much stronger and powerful now. And much more like, you know, this is what I want, and this is what I will pursue; making my own choices. Before I would never have done that, I didn’t dare… No, I didn’t dare and I started doing that and standing up for myself more. I have decided myself what I will and won’t do. And before, I have never had that: until two years ago, I have never had that.’ (P8)

(f) More expressive & assertive & aggressive in social interactions. Many participants report that in social interactions they speak out more what they think of things, and generally express themselves more. They also stand up for themselves more, are more assertive. Some also report being more easily annoyed by other people, and being more irritable. Three participants were more aggressive, mainly in traffic. The latter was in both cases pointed out to the participants by others: they had not realized it before.

Well, your mood improves, right, you become more cheerful–so yes, I started talking much more, I became much more assertive, I should say. I really stood up for myself much more, sometimes even a bit too much.. (…) If now the battery is switched to the right settings, than I already warn them at work: ‘Yes, sorry, I can’t help it, but I might be a bit witty.’ Yes, than I also say literally how I think about things, and then.. Yes, that does not always come across well, of course. You can sometimes think everything, but you cannot say everything. But then I actually say everything I think!’ (P6)

‘I just walk up to anybody, I shake people’s hands, I am more spontaneous, I dare to stand my ground by now. And if I disagree with something, I will simply say so. Yes.’ (P10)

#### (2) Life world & social interactions

(a) Doing things. When participants dare to do things that they used to avoid, their life world changes too. Suffering from chronic and severe OCD, the life world of most participants had gotten very small. Many participants hardly ventured out of their house. Some participants for instance did not dare to go to any other toilet than their own, which alone already shrunk their radius of action considerably. Being less anxious and more careless, participants now often do undertake things; ordinary things, such as doing groceries, and meeting people, or taking up a hobby.

‘I used to be terrified of blood; I didn’t dare to sit on chairs and everything.. and public toilets and those kind of things. And now that all goes so well. So that makes me feel more human. Because I was really restricted by those things. Sometimes so badly, that I didn’t crawl out of the door anymore.’ (P17)

‘I used to avoid a great many things that I now do do. But I used to avoid them in order to just not have to wash my hands 1500 times afterwards. And now, yes, no I wash my hands maybe 40 times, but that is two minutes. And then you are more inclined to do things. (…) So those are everyday, small things at the office, and at home. Yes, it’s just nice that you don’t have to wash your hands for every trifle.’ (P16)

(b) More engaged & interested. A large majority of the participants (14) were more interested in the world around them, and more engaged. Several participants mention that they are more interested in listening to the news, to know what is going on in politics, and public debates. But also on a smaller scale, participants recount being more open to the world around them. One participant for instance gave the example that after the DBS had been switched on again (after being switched off for some days for research), he immediately noticed the nice shoes of the psychiatrist and even made him a compliment. Other participants mention that they are very happy and proud that they are now in the position to help others, which was not feasible before.

‘I always used to have that, that I reacted spontaneously at things.. Yes, and when I got sick, that got less; the interest gets less. But I always used to have that, that I am compassionate, and caring for others. So I have always had that, but it had been toned down, and now it’s back again.’ (P15)

For most participants what they were interested in stayed the same; they picked up their old interests from before their OCD had gotten out of hand. Two participants, however, mentioned a change in interest: one participant joined a walking club, although she had disliked making walks before. Another participant used to do a lot of sports, but now none at all. One participant (P11) became *less* interested in the world and the people around her: she did not care so much anymore about the things she used to care about, was less curious and less inclined to delve into things, as she used to do.

(c) More part of the world. Many participants compared having OCD to being imprisoned, to being trapped inside their heads. And even though all participants still have at least some remaining compulsions, they commonly express that they now feel more part of the world again.

‘You have lived your life aside of the world, all those years, decades. I have had days that I was so turned inwards, continuously doing my rituals, and then I was lying in bed and heard at the radio that it had been a rainy day. And I thought to myself, was it rainy today? I had no idea. And your gaze has passed the windows of your house a thousand times, but you don’t notice it, there is no outside world anymore. (…) But then I caught myself really listening to the news and I realized this might be a sign of a small, beginning, change. You start to read the newspaper, and you start to talk to people, say what they think of the financial crisis, or something. Yes, it becomes more and more your own world.’ (P4)

‘A large part of your daily life now gets filled with contacts, with talking to people, with making plans, and seeing how you can make them work. That gets pushed away or something by those compulsions. Again; I am not there yet. One third of the day [is still filled with compulsions] may seem a lot, but for me, it is already a sort of.. well, you get out of your cage, or something. Yes. I used to be completely trapped in those compulsions.’ (P4)

On the other hand, several participants also note that the world they now find themselves in, has changed considerably during the time they lived in the ‘bubble’ of their illness. Some find that the Dutch society has become tougher, offering less provisions for those who are less well off. In this respect, some feel less at home in this changed society.

(d) More time for social contacts. Changes in the life world of participants of course include changes in their social interactions; affecting not only themselves, but also their partners, family, and friends. Almost all participants have more time and ‘space’ for social contacts: to the extent that they are less distracted by being occupied with their compulsions, they can be more present and attentive. Some participants remark that their friendships have deepened. Several participants report that they had to build up their social network again, after a long period of relative isolation. This may be quite difficult. As a participant remarked: ‘Now I have time for meeting friends and I find that I have none.’ (P12). Another participant (P11) experienced less interest in others, to her own regret.

(e) Less dependent upon others. As the impact of the OCD diminished, participants were also less dependent on others. They did not need others to double check the windows, doors, and gas, or to affirm that they had indeed washed their hands, or to reassure them that there was nothing to be afraid of. They also needed less help with the tasks of daily life.

(f) Impact of personality changes on social interactions. In so far as participants changed, this naturally affected their social contacts. Many participants (13) have become more expressive: they for instance make more jokes, are more talkative, and spontaneous. Someone reported being more expressive also in a bodily way; giving friends a hug, or a slap on the back. A large part of this group (10–11) had also become more assertive: they stood up for themselves more, and let themselves be heard when they disagreed with things. This sometimes leads to conflicts, and a few participants lost old friends as a result.

Yes, it feels a bit strange if you never were this way; you have always adjusted to other ideas, and now, all of a sudden, you don’t do that anymore. The people in my surroundings really feel like… you just fall outside their expectations all of a sudden… (…) Yes, and that is not always possible. Relations are based on pretty fixed givens, and if that suddenly changes radically, than it is questionable if that relation can keep on existing.’ (P11)

Some participants remarked that they had become less sensitive to hierarchy: they were less impressed by the social status of their collocutors. Interestingly, for two participants this was an immediate effect of the right DBS settings: they all of a sudden regarded the doctors around the table as ordinary human beings. Five participants also note that they are more easily irritated and annoyed by others, and had less patience. Two of them felt that they were a little too snappy to their liking. Another participant remarked that he thought this was an indirect effect: because he was less dependent upon other now, he could permit being more direct and assertive.

‘I used to be terrified to just get fired. I always used to worry about that; thinking ‘if only I don’t get fired’. And that’s why I always said yes, even to the most impossible assignments I said yes–I did not dare to say no. That was often very difficult, and it often brought me in a difficult position. Because I always said yes, I often got saddled with, to be honest, really annoying work. Like: let Adrian do this, he will agree, go to Adrian, he will deal with it; he will say yes anyway. So it was taken advantage of, that was what it was. You only realize that afterwards. (…) But then I had the operation and then with an assignment I suddenly said to my boss: I won’t do it. All of a sudden, I said no. And the strange thing was that I also was not afraid to get fired. (…) Even more so: I thought: I will fire you–even though I was the subordinate one. I didn’t worry. I worried about nothing anymore. And the people around me had to get used to that.’ (P14)

(g) Relationship with partner. Several participants report that they had difficulties in their relationship after DBS treatment. Their partners had to get used to their sudden changes in behaviour; in being confronted with ‘a different person’ as one participant described it. Moreover, many participants experience ups and downs in treatment, which makes them less predictable to their partners. Whereas participants themselves were generally happy with the changes, several noted that their partners were considerably less enthusiastic.

Before the operation I was Adrian 1, and now I am Adrian 2, so to speak. (…) It was very difficult for my wife, and also for my children. For my wife it was very difficult to be confronted with Adrian 2, because I reacted differently to most things. (P14)

A few participants had met their partners after the effects of DBS had already set in, and did not have these difficulties. One of them felt that being in a relationship had not been possible before DBS.

#### (3) Characteristics of the interaction

Not only the participants and their (social) life world changed, so did *the way in which* they interacted with the world and with others. In other words, their mode of engagement changed.

(a) More outward directed. First of all, participants were generally more directed at their surroundings instead of their inner worries.

‘Yes, I can pay attention somewhat better, so to say. Having conversations more easily, or yes, at school that it goes better. Somewhat better at focussing on the task at hand, or at the music [while playing in a band]. Or just watch a comedy for half an hour, say. Because normally I always used to go to the toilet; to the toilet, or washing my hands. Or I got so distracted with my thoughts and then I could not follow the story anymore, for example. So that has improved.’ (P3)

(b) Doing things without thinking. Another remarkable change that participants reported, was that they started doing things unthinkingly, that they used to pay conscious attention to. Compulsive rituals need to be performed with utmost attention, and in case of distraction, participants had to start all over again. Now, they caught themselves doing things automatically. Or they even realized only afterwards that they had apparently done something, such as closing doors and windows, without their usual concentration and rituals. Some participants became uncertain, wondering whether they had actually done things in the ‘right’ way. Most participants, however, welcomed this change, and tried to hold on to and expand it.

‘I can now listen to music while performing compulsions. (…) [Before I couldn’t] because I had to do them so carefully, so consciously that I should not be distracted, by anything. Because yes, you will unintendedly listen to the lyrics.. (…) Now I can because I have become more nonchalant; I can leave it more. And of course I know, it does not have to be done so consciously; if I only did it roughly, you know. It is still compulsive behaviour, but it is no longer that energy draining and that consciously. (…) Yes, that is the case with much more compulsive behaviours. (…) Yes, you notice that, yes. Yes, so it is not yet that you can skip the compulsions, but you still need to do them, but it does not have to be done so consciously. Yes, you trust somewhat more in your automatic responses, let me put it like that.’ (P18)

(c) Flexibility. Some participants (4) find that they have become more flexible: both in their thinking and their behaviour. They are better able to switch between tasks, and are less annoyed when they are being distracted during a task. Another participant however had more difficulties with multi-tasking, but also mentioned that before DBS she might not have taken all these things upon her in the first place. A few participants (3) were more chaotic, and had difficulties planning and organizing.

(d) Concentration. Five participants note that they are better able to concentrate. A few of them suggest that this is possibly due to the fact that they are less distracted from obsessions or compulsions. Two others, on the contrary, have more troubles concentrating: they find it hard to focus, and are more easily distracted.

#### (4) Existential stance

With the existential stance, we refer to the fact that people relate to and take a stance on themselves, on what they experience, and on their (social) world. The existential stance thus refers to the ‘second-order’ relation to one’s first-order experiences. This, typically evaluative, stance plays an important role in the overall experience. Take for example the experience of increased libido: to some extent it is the person’s stance on these experiences that determines whether or not it is regarded as a side-effect. Or take the experience of increased assertiveness: people need to relate to and assess whether this fits them or not.

(a) Attitude towards changes. With so many different changes, and such a variety of experiences, the participants’ attitudes not only vary between participants, but also depending on the kind of change. On a very general level, we can say that many participants tend not to worry so much about the changes they experience: not about the welcome ones, but neither about the unwelcome ones. One participant for instance notes that he has become sloppy, but, to his own and his family’s surprise, he does not care about that at all. In cases where participants did worry about some of the changes, these worries were typically directed at the effects of treatment on their social interactions, for instance the loss of friendships because of their increased assertiveness (cf. (1f) and (2f)).

Several of the changes as listed in the first domain can be regarded as changes in personality. How did participants relate to these changes? Did they feel more, or less themselves? And how did they themselves assess this question? This is a highly relevant and much debated topic, especially in the field of neuro-ethics (Cf. [43,44,45]). For reasons of space, we will not get into this issue here, but discuss it in a different article.

(b) Attitude towards compulsions. All participants had at least some remaining compulsions. But often, participants noted that the compulsions felt less burdensome, and they remarked that they could let it go more, and were less stressed while performing them.

‘Well, that was also very funny: when I went there [to the hospital], by car, I had the radio turned on. And on the way back [after the DBS was turned on], I was whistling along with some songs. So that is.. than I am still all the time preoccupied with compulsions and anxiety, but at least I have.. yes, now I can also be cheerful. Although being cheerful like that, it could be, I think, that I more act that I am cheerful, than that I really am, but that cannot… if it [the DBS] is off, than that is not possible.’ (P7)

Besides, several participants noted that their attitude towards having compulsions had changed: they were no longer worrying so much about that, but rather accepted that some compulsions would probably always remain. They were less inclined to worry when they had ‘bad days’, and could trust more that it might be better the next day. Another participant (P12) remarked that after having tried every treatment, and having hoped for a total cure, she now tried to accept that her OCD would never completely vanish.

(c) Attitude towards DBS. For all participants, treatment with DBS was their last resort after a history of insufficient results with other treatments. Most participants were in untenable situations, and some of them were so desperate that they considered suicide. As several participants explained, they felt they had no other choice than to try DBS. For some participants, DBS did not bring what they had hoped for: they felt better, and less anxious, but still spend many hours on their compulsions. This was of course quite a disappointment.

‘I would just want more [than 20% reduction of complaints]. (…) In my household, so to say, I can just slip up, whereas I couldn’t do that before, and now I can. I am already happy with that, but I would rather also have things for myself, so to say, be easier on myself. Yes. And yes, I still need to wash my hair every day and preferably two or three times even. Yes, that is not good. And I would rather want that I could do it only one time with shampoo, one time with soap, and that I would than be done. But that is not case, it is all compulsive. (…) And it just takes a whole lot of time.’ (P15)

Still, all the participants preferred to have DBS. A few participants worried about their dependency on the hospital, for changing the DBS settings, and for replacing the batteries. One of them remarked that he could not emigrate to his son, who lives abroad, because of this dependency. The majority of participants however, did not experience such a dependency. A more common worry has to do with the dependency on the device itself: what if it would somehow stop working, or what if electricity would fail and the rechargeable battery cannot be charged? With regard to having a device implanted in their bodies, participants did not think about this, or feel awkward about it, not even those participants who had had troubles with the leads or the battery.

But this [the DBS] actually feels so… natural. As something that, a process that gets regulated. (…) And electricity, I mean, that is also just in your brain, so it is actually a kind of body’s own thing, and yes, in some way it does not feel artificial to me, or anything. Yes, I don’t know, I can imagine that if you have for instance somewhere, say a screw in your hip or anything. Of course it is not something with which you were born and that has been there or developed there naturally, but at a certain point it becomes… I don’t know. (…) I do not at all feel like I am a sort of ‘bionic woman’ now, or anything.’ (P1)

Some participants did worry about what others might think of them, and were afraid of negative reactions.

(d) Relating to past & future. Once participants feel better, and are no longer continuously absorbed by surviving, there is room to reflect on both past and future. Participants start making plans again, looking beyond the next hours. Reflection on the past is often painful: some participants mourn over the lost years and the missed chances. Others avoid to look back at all, because it only makes them sad to realize how parts of their lives have been destroyed by their illness.

‘Yes, sometimes I say; I have actually simply thrown 20 years of my life in the trash bin. But well, there is also someone in our group who is 65, who says: 50 years of my life have been thrown in the trash bin. That is a different story. That is also possible. So in that sense, I am still on time. (…) And I think that therefore my urge to do that study is so strong. It seems a bit like making up for things, or something, I don’t know.’ (P13)

‘It [the OCD] is so bad–especially if you were constantly occupied by it, like I was. You were only rarely freed from your anxiety and compulsions. Recently, I looked at a photo album of our holidays from years ago, and then I saw on every photo that I was bothered by my anxiety and compulsions. As I just said, you can tell that from the eyes (…) And then I was so shocked (…) Then I looked at those other albums, I looked at I don’t know how many of them, and then I reached the…yes, it made me very depressed, let me just put it like that. I only then fully realized what an awful life I had had.’ (P14)

(e) Experience of freedom. A large majority (14) of the participants felt more free. When asked about their experience of freedom, participants mentioned all the things that they could now finally do, like studying, or taking care of a sick parent, and how gratifying that is. Others also mention that they have become less dependent upon others. And many participants regard freedom in the light of being freed from their compulsions, and regaining control over their own lives. (See [46] for a more elaborate study on the experience of freedom of DBS treated OCD patients with similar findings.)

‘Well look, back then, before the operation, you had the idea like there was nowhere I could turn; I have to do what the dictator, the compulsion, decides. And that was, say, all hours you were conscious. If you were sleeping, yes, you cannot control your dreams of course, then still you had the inclination to… then I could sometimes wake up in the morning, exhausted from all the things you had been tidying and cleaning [in your dream].. But say the 16 or 17 hours a day that you were busy like that: yes, you were trapped. And now I can.. I can go to a cafe terrace. And then still I am… See, if I go walking (…) then the walking itself is relaxed, but still, unconsciously, I won’t walk into dog shit somewhere. (…) But all that used to be impossible. So you experience more freedom, certainly.’ (P18)

(f) Reorienting one’s life. The compulsions had determined the lives of our participants, and now, for most of them, there is (some) time and room again for engaging in things. For most participants, this naturally filled, but others experienced more difficulties. They sometimes feel: ‘and now what?’ (P11). As one participant (P12) notes: ‘Now that I have time for doing things with friends, I find that I have no friends anymore.’ Several participants note that they need to find out who they are: ranging from finding out which film genre they prefer and which hobbies they might like to what they want to do with their lives.

‘It is no longer only the compulsion that is in the foreground. I really feel that I am now more Lea. That is also pretty intense, for who is Lea? I have put her away for years, you know. Like I said; always that mask, and always the compulsion that would be in the foreground; it was only about that compulsion. That was the only thing that my life revolved about: compulsions. Yes and now I am looking.. I am doing volunteer work, I am going to calmly find.. you know, what do I actually want: do I want to work, or rather not, how will I do that? How will I spend my life from now on? That is what Lea wants, and not what the compulsions want.’ (P8)

Several participants also pointed out that the world had changed during the time of their illness, and that they sometimes had difficulties with getting used to the ‘hardening of society’ (P5) (cf. (2c).

### III.B. General course of effects

There are remarkable differences in the **course of effects** of treatment. Roughly, we see three groups: first of all, there are patients who experience immediate effects of DBS, either right after the device is switched on, or after finding appropriate parameter settings. Secondly, there are patients who experience changeable effects: there are weeks in which their symptoms improve, followed by weeks in which all effects are lost again even though the DBS settings remain the same. And lastly, there are patients who unfortunately experience no positive effects of the DBS, at least not on their compulsions. Some of these patients do feel slightly better; they are in a somewhat better mood. With some of these non-responding patients (‘non-responding’ both in terms of symptoms as well as in terms of their broader phenomenology) turning off the DBS makes them feel even worse than before treatment [47]; an effect which does not seem to disappear over time. Besides, all participants experience relapses in case of stressful events in their lives.

**The order of the effects** shows a considerable consistency, at least for those participants who profit from DBS. First, when good settings have been found, there is the experience of the anxiety going down, and the improvement of mood: feeling lighter, happier. Then spontaneous effects occur: participants simply forgot to perform some of their rituals and compulsions. For instance, they realized only later that they did not check the windows or washed their hands after touching something. Partners or family members often noticed these changes before the participants themselves. The third phase consists of working on the compulsions. With help from CBT, participants train to gradually reduce their compulsive behaviour. As one participant said:

‘You have to do a whole lot yourself. It is not–in the media it is sometimes portrayed as a magic wand. Yes, it is on mood. And also somewhat on obsessions, but these compulsions… for these are the bars that surround you.. Transparent bars, and you will have to cut each and every one of them yourself. And the one is… And I see at the peer-meetings that for some that requires an enormous effort. Not so much anxiety, because the anxiety does not arise, but you have to push yourself to do it. And if you want to achieve that 80%, 85% [reduction of symptoms] … yes, you will have to work very hard for that.’ (P13)

The last phase can be summarized as the re-organizing of one’s life. Now that their life is no longer dominated by OCD, people need to re-orient what they can and want to do with their lives.

The **effects of DBS on obsessions and compulsions** seem to be threefold. First of all, treatment has an (often spontaneous) effect on what *triggers* obsessions and compulsions. Situations that used to evoke obsessive thoughts or compulsive rituals, no longer do so. Participants often only notice these changes after their partners and/or family has pointed them out. Secondly, in case people do perform their compulsions, they can be quicker: it takes much less time before it feels right:

‘Yes, you don’t get stuck that much, you know. I always say: the penny drops. Yes, well, that is really appropriate in this case. Because yes, the signals.. what you see.. what you see with your eyes if you lock a door, yes, I see that too; with me it just does not arrive where it should arrive. (…) You don’t get any confirmation in your brain. So you get stuck: you keep on pulling that door, and pushing it… you keep on turning the key, although your eyes see.. Sometimes I would very, very consciously lock the door–and still the penny does not drop! And then [with the DBS] it went a lot easier. Yes, it went much more easily. I could let things go much quicker.’ (P6)

Thirdly, many participants report that they feel less stressed or tense *during* the performance of their compulsions.

### III.C. Side-effects

Participants also reported several side-effects of the DBS treatment. Firstly, with regard to *surgery*: some participants reported a straining feeling from the leads being too tight (2), leads getting out of place (2), or the battery moving in the chest (2). In these cases, another surgery was needed to remedy these problems. With one participant, the electrodes were not implanted in the right brain area and had to be replaced. Secondly, with regard to the *device* itself, three participants reported feeling electrical pulses around the battery (leakage current), or feeling the battery. Thirdly, *stimulation-related side effects* that were reported, include problems with (short term) memory (9), word-finding (6), concentration (2), feeling tired (2), and weight gain (5). One participant reported feeling dizzy, having a disturbed sense of time, being disoriented, being less creative, and having an impaired control over her movements. Another participant reported a transient craving for alcohol. Several participants (7) experienced transient hypomanic phases after setting changes of the DBS (usually an increase in voltage), lasting approximately two days. Most people enjoyed these days and wished it would last longer, although some (2) also thought their energy and high spirits were a bit ‘too much’. An increase in libido was also mentioned by five participants, but they differed in their evaluation of these changes. Three of them regarded their increased desire as a resurge of their natural inclinations, that had been suppressed by their OCD. The other two participants however, felt it was not normal, and not fitting. For more on side-effects of DBS in the nucleus accumbens for OCD patients, see: [11,29–31,33].

## Discussion

### IV.A. What is new?

Several of the experiences that our participants report have been described before; notably the lessening of anxiety, the improvement of mood, and increased impulsivity. These are of course highly relevant and striking effects. But our results show that there are much more changes following DBS treatment, such as an increase in trust, self-reliance, and self-confidence, a more unreflective mode of engagement, and a more careless stance on things. These changes are not covered by any of the presently used scales, such as the Y-BOCS, the Hamilton Depression Rating Scale, the Hamilton Anxiety Rating Scale, or the WHO Quality of Life scale. It is important to note that these are *general* changes, and that their impact is not limited to obsessive and compulsive behaviours only. In order to get an adequate overview of the *general experiential effects* of this new treatment, these changes too should be included. What is missed by the existing scales is precisely a grip on the global changes that patients experience. But if we wish to understand what DBS actually does, the experiences of patients are a crucial, if not the most fundamental, aspect to take into account. Besides, the experiences of patients may help to better explain the findings that have been obtained by using standard scales. For instance, as mentioned in section I.D., it was found that for OCD patients who were treated with DBS their scores on the short version of the WHO Quality of Life Scale improved in all domains, except for the social domain [39]. This finding could perhaps be explained by the difficulties that some participants experience in their social interactions now that they have changed considerably (cf. (1e), (1f)). Also, there may be a painful discrepancy between the desire for more social contact and the lack of a social network due to the long history of illness (cf. (4f)). And finally, an important advantage of a qualitative study such as ours is that an overview of possible experiential changes enables clinicians to better inform patients beforehand on what they and their loved ones can expect to happen.

### IV.B. Interpretation of coherence of changes: from anxiety to reliance

Participants thus report many changes, on many aspects of their lives. The lists of the experiences in the previous section may be a bit overwhelming. So how could we make sense of all these changes? Are they in some way related? In this section we propose a specific interpretation to get a better grip on the wide variety of effects. We suggest that many of these changes can be seen as *different expressions of the same process*; in which the experience of anxiety and tension gives way to an increased basic trust and increased reliance on one’s abilities. This increased trust and reliance are manifested by an increased outward-directedness and openness to the world, and increased spontaneity in the way of acting and reacting. Instead of doubting, worrying about all things that might go wrong, and meticulously controlling every detail, participants start relying on their abilities, assuming that things will work out, being more direct and careless. In general, we suggest that the way of being in the world of these participants used to be characterized first and foremost by *anxiety*, whereas following successful treatment, anxiety has given way to more *trust* and openness.

Looking again at the four different domains of changes, the diminishing of anxiety was a prominent experience in the domain of person and self-experience. The improved mood may–at least to some extent–be a part of the same process as well: being less anxious and less worried implies feeling better. The extent to which anxiety and mood are connected, seems to differ amongst participants. This may depend on whether or not the sombre mood before treatment was a *secondary* effect of a life with OCD or of being continuously tense or anxious. If their sombre mood was secondary, than it can be expected to alleviate with the lessening of OCD symptoms. Participants also showed an increased trust in their abilities. This trust and self-reliance seems to be the basis for being less reflective and reacting more spontaneously and impulsively. Being more self-confident, participants say what they think, are more assertive and direct. Along the same line, participants also report being more easily annoyed, impatient, aggressive, or disinhibited. This seems to be a gradual phenomenon with on the one extreme an anxiety induced inhibition and (hyper)reflectivity; being overly controlling and deliberative [8]. Becoming less anxious, and more unreflective than leads to a welcome increase of openness, spontaneity, and directness. But becoming even more loose, means acting thoughtless, rash, and potentially rude. Whilst being more assertive will be a great gain for those who have let others walk all over them, if this turns into the other extreme of having a short fuse, or becoming inappropriately aggressive, it is too much of a good thing. Participants report both being more spontaneous and direct in social situations, but some participants also find they are less careful and sensitive to the needs of others. For hypotheses about the neurobiology of these effects on mood, spontaneity, and assertiveness of DBS in the nucleus accumbens, see: [19,29,33].

With regard to the second domain, the (social) world of the participants, we can see that their world opens up and becomes bigger. Participants are no longer confined to the compulsions that HAVE to be done NOW, and are thus able to see a larger range of possible actions, both immediately and in the future. They note that they see perspectives again. We have described this difference before in terms of different ‘*fields of relevant affordances*’ [42,48]. Affordances are possibilities for action that the environment offers you [49–53]. A cup affords drinking from, an apple affords eating, a keyboard affords typing, a person affords interaction. In any situation there are always multiple possibilities for action, comprising a field of affordances. Depending on what is out there and what your needs and concerns are, some of these affordances will be more inviting to you than others: when you are hungry, the apple will be more salient to you than the keyboard.

**Fig 1:**
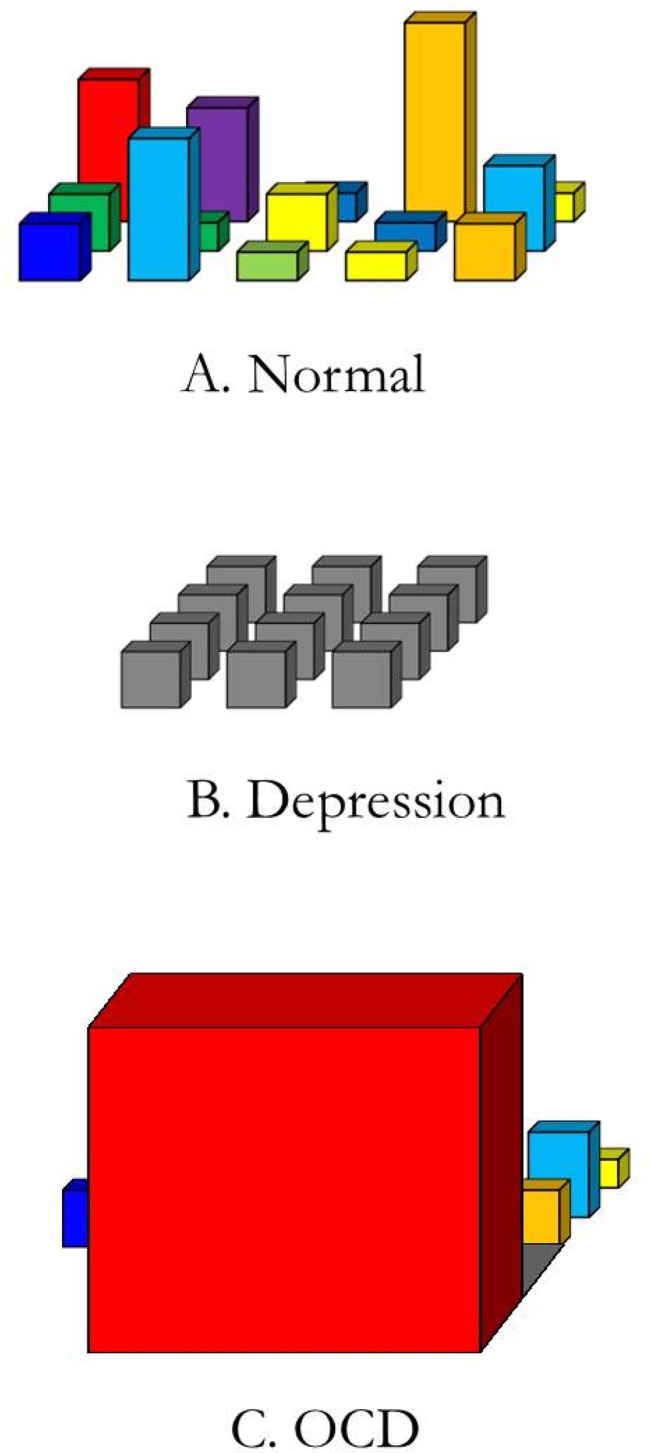
Different fields of relevant affordances (Improved figure, reprinted from: [42]).

Fig 1 gives a schematic depiction of different fields of relevant affordances. The ‘width’ refers to the range of affordances or the amount of action options that one perceives. The ‘depth’ of the field refers to the temporal aspect: one not only perceives the affordances that are immediately present here and now, but one is also (pre-reflectively) aware of future possibilities for action. That is, one may already anticipate the affordances on the horizon. Lastly, the ‘height’ of each of the affordances refers to the relevance or salience of this particular option. The different colours refer to variations in affective allure: something may be relevant because it is dangerous, or rather because it is highly attractive. It is a dynamic field: to the extent that either our concerns or the environment changes, the field of relevant affordances changes too [42].

For patients suffering from OCD, their field of affordances is narrowed down to only the compulsions that require all their attention and overshadow any other possible actions (cf. Fig 1C). Completing the compulsions has immediate priority. The field of OCD is very much a field of fear or anxiety: fear narrows down the focus to just what is feared, and fear takes primacy over all other perceptions and actions. If, for instance, a fire would break out, this would immediately draw your complete attention—and all other possibilities for action (drinking your coffee, reading an article, chatting with someone) lose their relevance. Compulsions are like an outbreak of fire in this sense. For some people, all the compulsive rituals they need to do, can seem equally urgent: leading to an overwhelming situation of permanent demand. Not knowing which fire to extinguish first, so to speak, people may find themselves paralyzed by conflicting urges.

When treatment is effective, and the anxiety diminishes, the extreme focus on the here and now gives way to a more open view. Participants notice more possibilities for action, both now and in the future. They are able to plan ahead. Some participants mention that they now have a better overview on things:

‘Then I came home [after the DBS settings had been changed], and I cleaned the whole house in one day [which she would normally be too scared to do]. And then my partner came home and he said: ‘what is the matter with you?’. I said: ‘Yes, I am doing terrific.’ And then you see that it [the DBS] works on much more than just anxiety: I had lost the anxiety, I had a tremendous amount of energy, and I suddenly was confident about everything. So you feel much better in terms of mood, and I also suddenly had the overview again which I lack when I am in an anxious period: like ‘oh, if I do it like this, then I can right away take up on that, and then I will tidy up there and move that over there. And I will first do this, and then that.’ And this overview is something you completely lack when you are trapped in the compulsions.’ (P9)

With regard to the third domain, the mode of engagement, we have already pointed out that participants are *more outward directed*. Moreover, they start doing things unthinkingly, rather than deliberatively controlled. Such a spontaneous, unreflective way of acting requires that one relies on oneself and one’s capacities for acting adequately. Instead of the convulsive focus, participants become more flexible in reacting to other possibilities for action, and thus better at switching between tasks. It seems that participants are more triggered by relevant outside events (‘bottom-up attention’), rather than deliberately steering and controlling their actions (‘top-down attention’) [54]. This too seems to be a gradual phenomenon: the increased flexibility can also tip over into being more easily distracted, and even chaotic, as some participants experienced.

Lastly, the fourth domain of changes with regard to the existential stance also fit with this general pattern of increased trust and openness. Those participants who profit from DBS, are no longer pre-occupied by immediate survival, which opens up the possibility to step back and relate to one’s past and future. Besides, the more nonchalant attitude, relying more that things will work out, stretches out to the way in which participants relate to themselves, their remaining complaints, and their future: they are more optimistic and hopeful. They see less problems in things–also with regard to the way they have themselves changed through treatment. That this sometimes leads to tensions with their partners and family, indicates the necessity for therapists to assess whether this relaxed attitude is appropriate, or perhaps a little too careless.

Summing up: we suggest that various changes that participants report are indicative of the same process of increased trust and reliance on one’s abilities over anxiety and tension.

### IV.C. Strengths & limitations of this study

The main strength of this study is that it offers an investigation of patients’ experiences without being pre-structured or pre-filtered by psychopathological scales. Since the effects of DBS treatment are more global than can be measured by the Y-BOCS, it is important to get an overview of the general picture of the changes in experiences and life-world of OCD patients who are being treated with DBS. So far, information on the effects of DBS on patients’ experiences is either limited to the use of scales, or else consists of case reports (cf. Section I.D). We have interviewed 18 participants, which is in general a good sample size for a qualitative study [55,56]. Our use of purposeful sampling rather than convenience or random sampling helps to ensure that we cover the greatest variety of experiences. Besides, all participants are treated at the same hospital and underwent the same procedure: their DBS target brain area was the same, as was their treatment protocol.

What should be kept in mind is that there was no control group, and so our study does not distinguish between effects of recovery *as such* and effects of recovery *through DBS specifically*. It is likely that any means that would succeed in a considerable reduction of anxiety, would lead to several similar changes as the ones described here. What *is* specific of DBS treatment compared to other forms of treatment for OCD is the potential speed of changes. In case of rapid response, the transition of illness to recovery is more abrupt, which might lead to more vivid experiences of these changes, potentially making them better visible and articulable.

Secondly, the distinction between primary and secondary effects of treatment is also not straightforward. It seems that DBS reduces the experience of anxiety and tension, which may in turn (and in combination with therapy) lead to other changes. For instance, being less anxious may reduce the need for compulsive behaviour, which means that there is more time available for other activities, such as social events or work. These activities in turn may have a positive effect, by providing an external structure, or by feeling less lonely, or more confident. Having more self-confidence may then help to try to further reduce the compulsive behaviours. In this way, feedback loops and positive spirals may emerge. This complex system of experiences, reactions, and outside influences, makes it difficult to tease apart the exact role of DBS. Besides, seven participants continued to use medication.

A third issue concerns possible placebo effects. Several studies [11,24,26,30] included a (double) blind on-off phase, reporting diverging ‘placebo effects’. Recently, a randomized controlled study has been published in which patients with treatment-resistant depression either received active tailored stimulation in the ventral capsule/ventral striatum, or merely sham stimulation [57]. Remarkably, they found that after a 16 week period there was no significant difference in response rate between the two groups (3 out of 15 were responders in the active group, versus 2 out of 14 in the control group). This suggests that the effects of treatment may be more of a placebo effect than previously assumed: that is, other factors than the stimulation itself may play a bigger role. These could include factors such as the impact of being selected for an innovative, expensive form of treatment, the high hopes that this may stir, the regular monitoring meetings, being treated at university hospitals with highly qualified staff, and the attention and care patients may experience. Moreover, patients agree on undergoing an invasive surgery: many of our participants felt that this was the last thing they could try, and mustered all their energy to make this one more effort to fight their disorder. Summing up, in the present study it cannot be indisputably established what are the effects of DBS specifically as opposed to (a) recovery as such, (b) CBT, (c) secondary effects, and (d) placebo effects.

Another factor to keep in mind is that our sample is not statistically representative of the 42 DBS-treated OCD patients at our department. The given numbers and percentages thus cannot be extrapolated to the larger group of patients at our department, let alone to the larger group of OCD patients treated with DBS worldwide. This is in line with our aim to get an overview of the *variety* of changes that patients experience–not to provide an *exact quantification* of the occurrence of each of these experiences. For our purposeful sampling, we relied on the feedback of the therapists to select a most diverse range of patients to participate. Still, we cannot exclude the possibility that we have missed out on important experiences of the patients who did not participate (for instance because their experiences were unknown to their therapists). With regard to the differences between our participants and the larger group of DBS treated OCD patients at the AMC, it should be noted that there were more responders in our sample: 11 out of 18 participants met the response criterion of 35% reduction on the Y-BOCS (61%), whereas in the total group 20 out of 42 patients are currently responders in this sense (47%). This difference arose mainly because several patients who did not profit from DBS were still severely ill and participating in this study would have been too much of an extra burden for them. On the other hand, some other people who had almost completely recovered from OCD were also not included: they did not want to participate in any research anymore, because they would rather just go on with their lives. In other words: both extremes in response (extremely weak and extremely strong) were lacking in this study.

As a last limitation, it should be noted that participants were interviewed 6 to 81 months after surgery. This means that participants need to rely on their memory with regard to the early changes that occurred during treatment, with the ensuing possibility of distortions. Although the most impressive changes are likely to be remembered, it may very well be that some nuances got lost.

## Clinical Implications for DBS Treatment

What are the implications of our study? First, with regard to the **clinical procedure**, participants indicated what had been helpful to them and also offered several concrete suggestions for improvement of treatment. For the first phase, participants stressed the importance of being informed extensively beforehand. In particular, three possible scenarios need to be discussed beforehand: (1) no effect; (2) fluctuating effects; (3) good effects—and how to proceed in each of these cases. Many participants pointed out that it is crucial to explicitly warn patients beforehand that they will probably never get rid of *all* their obsessions and compulsions; that some symptoms will remain—even in case they would actually profit from DBS. Besides, even though DBS can be a major enabling force, patients should be made aware that they still need to work very hard to reduce their obsessions and compulsions (for instance as part of CBT). Such explicit information could prevent patients from having unrealistically high expectations of DBS treatment. Patients and their partners and/ or family should also be informed about potential changes in personality that may occur, and about the potential impact of treatment on partners and/ or family as well. Participants also indicated that it would be helpful to involve partners and/ or family during treatment in order to get a better picture of the patient, but also to support them in dealing with the occurring changes.

Several participants indicated that they would have liked to speak to someone who already had DBS treatment: both during the initial, informative stage, and also later on. It is after all a unique treatment, and some had felt a bit lonely facing the changes they experienced. At our department, special ‘peer-meetings’ are organized twice a year for patients to meet and exchange experiences. Many participants indicated that they profited from these meetings, but they would prefer to be able to exchange experiences on a more regular basis. Some suggested an online forum, or a ‘buddy system’ to have the possibility to get in touch with each other according to one’s own needs.

With regard to the later stage of treatment, our findings testify of the **importance of including CBT** or another form of psychotherapy within DBS treatment, as has been argued before [58]. As we already noted, participants remarked that the DBS enabled them to engage in CBT. Before DBS, the anxiety had been too high, or they did not succeed in sustaining the effects of CBT. Importantly, this implies that patients should be prepared that the implantation of the device is the *starting point* rather than the *end point* of DBS treatment. DBS is not merely a neurosurgical treatment: it is rather a global treatment which encompasses the neuro-stimulator. In other words: the implantation of DBS may enable a process of recovery, but often a lot of work remains to be done by the patients. This includes working on reducing compulsions, but also more generally the need to re-organize their lives and to come to terms with how OCD has affected them. This process too may require support from therapists.

At a more general level, our findings indicate the limitations of the Y-BOCS as the sole **measure of success** of treatment. Several participants of our study are non-responders in terms of their Y-BOCS scores, but they do report that they profit from treatment. One ‘non-responding’ participant even says ‘the DBS has saved my life (…) it made my life bearable’ (P1). The Y-BOCS simply does not capture all relevant changes. For instance, from spending 16 hours to 9 hours on compulsions is a big difference, but it still gives the same score. The general level of anxiety and stress is insufficiently taken into account, as is the *experience* of the time spend on the compulsions. That is, it makes a huge difference if one feels continuously anxious and stressed during the performance of the compulsive behaviour, or whether one still performs them but without this tension and pressure. Besides, the Y-BOCS does not include a measure of how much people *avoid*. That is, if patients avoid many situations (going outside their house, for instance) in order not to have to do their compulsions afterwards, they will score lower on the Y-BOCS than if they would engage in such challenging activities. Simply counting the amount of time spend on compulsions cannot capture the invalidating effects of such avoidance. This lack of both avoidance and experience of tension is relevant not only because the Y-BOCS is the primary measure of treatment success, but also because it serves as the paramount measure of the severity of OCD as well. The selection of patients who are deemed eligible for DBS for instance depends on their Y-BOCS scores. In general then, the ‘objective’ characteristics, those aspects that can be counted by an external observer, cannot cover the whole story: the personal experience of this behaviour should be included as well. Just as persons’ experience of their world, and of themselves, and their stance on these experiences.

In line with this, the findings of this study demonstrate the need for a debate on **what counts as being a responder**: which changes do we consider to be relevant? When is DBS treatment justified? In case of P1 who still spends roughly the same amount of hours on compulsions, but feels much better, what should be decisive? The shear amount of compulsions does not seem to be a sufficient measure anyway. And if a patient feels better, that is a huge gain. On the other hand, it does not seem desirable if the situation is still as bad as it was before, only now patients do no longer experience it that way. One participant for instance reports: ‘I was still washing my hands with boiling water–only now [after specific DBS settings] I was singing while doing so. That’s crazy, right?!’ (P18). The difficulty is that one obviously wants patients to feel better, but that it does not seem right if this feeling better comes down to an artificial effect that is incongruent with the rest of their lives. This dilemma is of course not specific for DBS treatment, but rather concerns psychiatric treatment in general. It raises questions about what the appropriate aims for psychiatry are: treating symptoms only, or improving the general well-being of patients? And, in case of the latter option, how should patients’ ‘well-being’ be interpreted? These are fundamental issues that are nevertheless at stake in everyday clinical decision making, for instance when deciding on whether or not to continue treatment.

Another matter of debate is what should be the **unit of analysis** when it comes to determining the success of treatment: is that the individual patient or rather the patient in relation to her social environment? What if the patient is perfectly happy with the changes, but also loses her partner and the contact with her children? This may seem far-fetched, but in practice therapists have to deal with such considerations, for instance when it should be decided whether an increase in voltage as wished for by the patient, is indeed the best option.

## Suggestions for Further Research

Since DBS treatment for psychiatric patients is still relatively new, and does not involve large numbers of patients, much is still unknown. With regard to the phenomenological effects as discussed in this paper, it would be relevant to investigate to what extent these changes are *specific of DBS*. Would similar changes occur after successful CBT, or pharmacological therapy? Could it be that any means that would (drastically) lessen anxiety might lead to such changes? A related question is to what extent these changes are specific of DBS with *patients suffering from OCD*. Do similar changes occur in DBS treatment with patients suffering from other psychiatric disorders? Or with patients with movement disorders or Parkinson’s disease? There are several remarkable similarities between the experiences of our participants and Parkinson’s patients who are stimulated in the STN [59,60]. Parkinson’s patients not only experience a similar effect on anxiety and mood [59], but they are also more direct, more talkative, irritable, impatient, and expressive [60]. Besides, several studies have shown that Parkinson’s patients became more daring: following DBS, they take more risks and make more impulsive decisions [61,62]. Schüpbach and colleagues [60] also point to the common difficulties of their patients with their partners, and to the experience of a loss of aim in life. As they rightly note, several of these changes may be due to the abruptness of the improvement. Still, it would be interesting to investigate these similarities, and see if they may perhaps teach us something about the working mechanisms of DBS.

Moreover, the experiences of patients also raises the question what these changes imply with regard to the potential *effectiveness of DBS for other disorders*. That is, for OCD patients, DBS in the nucleus accumbens seems to be particularly suited for alleviating anxiety, tension, and overly cognitive control. The increased spontaneity and impulsivity following stimulation is a very welcome, correcting effect in case of OCD patients who rather exert an excessive amount of control over themselves and their actions [8]. However, there may also be conditions in which increased impulsivity would be particularly harmful. Of course, the initial connectivity in the brain will most likely be different in patients suffering from different disorders, and the DBS target in the brain may differ accordingly. Still, the experiences of OCD patients may encourage extra caution in these cases.

In the interviews, it came to the fore that there are often discrepancies between how participants and their partners evaluate the effects of treatment, with the partners being more negative. For instance, where participants are happy that they can engage in social events and speak out what they think of things, partners may feel that they have lost their feel for etiquette, and point out that this new behaviour puts pressure on relationships with friends and family. Moreover, the DBS treatment had an impact on partners and family as well; participants sometimes changed abruptly, or experienced very changeable effects, and partners found them unpredictable and were unsure of how to react. As one partner put it: ‘With him, a switch is turned, but with me, there isn’t.’ Future research could focus on *partners and family members* in order to get a better overview of the impact of treatment on their lives and to get a broader perspective on the situation of the patients as well.

Further research should also focus on potential *predictors of the effectiveness of DBS*. Are there any characteristics of the patient (e.g. kind of OCD symptoms, age of onset, occasion of onset, psychological characteristics, motivation, etc.) that indicate whether this patient will be a responder or not? On the basis of this study and our clinical experience, we hypothesize that DBS treatment is most effective in case of egodystonic mysophobia and egodystonic controlling compulsions. Perfectionism, ordering compulsions, and other egosyntonic symptoms seem to be less affected by the DBS (Cf. [11]). Another question concerns the role of avoidance: are OCD patients who are more prone to avoid perhaps less likely to profit from DBS? Besides, for some of the participants who are ‘non-responders’, the CBT part seems not to work out. Is that because their anxiety has not been sufficiently diminished, compared to the ‘responders’? Or could it be related to the participant’s attitude towards their compulsions, especially their inclination to ‘resist’ the compulsive tendencies? Maybe earlier successes with CBT, even if only small and/ or temporary, could be indicative of the effects of DBS on compulsions? Or might it be more effective for these patients to add a different form of therapy instead of CBT? These hypotheses would need to be tested. Since DBS is an invasive and also expensive treatment, in general more knowledge is needed to enable a better selection procedure.

Moreover, it would be very useful if there would be more specific (experiential) *markers* for the effectiveness of the DBS settings: which early signals indicate a stable good effect? Preferably then, a new scale could be developed that is sensitive to the changes that are missed by existing instruments–one that could also be used to determine the effects of DBS in the on-off phase of experiments. This is of course related to the question of how to define what we consider to be a ‘good effect’ of treatment. The Y-BOCS alone is not enough, but what needs to be added should be a matter of debate and further research. Furthermore, as we argued in the previous section, the Y-BOCS itself could be considerably improved by adding a measure of avoidance as well as personal experience of tension.

More in general, our study indicates the usefulness of investigating the relation between *quantifiable symptoms* as measured by several standard scales, and experiences of patients as described in this study. At least three aspects are insufficiently captured by present scales: First of all, what is missed is the personal *experience of* the symptoms: as several participants pointed out, an hour of compulsive behaviour feels very differently if it is performed while panicking or feeling very anxious, compared to the same rituals being performed without such tension. Secondly, people (evaluatively) *relate to* their experiences, to their illness, to their past and future, and to treatment. This existential stance has its share in the overall experience of patients. And, last but not least, the *interactions between patients and their environment* importantly contribute to the overall effect of treatment. In the best case, support from partners, family members, friends, and colleagues can contribute to the instantiation of positive spirals; helping patients to become more and more part of the shared world instead of being captivated in their own heads. Typically, the situation will be complex: with not only the patients themselves needing to adjust to the new situation, but their loved ones too. New interaction patterns need to be developed–and these will naturally have their impact on all participants. All in all, this suggests that ‘symptoms’, understood as the quantifiable and observable expressions of an illness, may not be so straightforward. And since symptom reduction serves as the main marker of treatment success, it would be worthwhile to discuss how to best fill in this concept.

## Conclusion

In this article, we presented the results of our study on the effects of DBS on the experiences of OCD patients. This study was motivated by the clinical experience that not all relevant effects of DBS could be captured by the standard psychopathological scales. In order to get an overview of the variety of changes that patients experience during DBS treatment, we conducted in-depth, semi-structured interviews with 18 OCD patients.

Apart from the previously documented improvement of mood, diminishment of anxiety, and increase of impulsivity, we also found changes such as an increase in trust, self-reliance, and self-confidence, a more unreflective mode of engagement, and a more careless stance on things. These are general changes, across the board; not limited to just obsessive and compulsive behaviours. We listed the main changes that participants reported, grouping them in four domains: (a) person, (b) (social) world, (c) characteristics of person-world interactions, and (d) existential stance. We subsequently discussed the general patterns in the course of the effects, and also listed the side-effects that participants reported.

In the Discussion section, we looked at the strengths and limitations of our study, pointed out what is new in comparison to previous findings, and offered an interpretation of the results. We suggested that many of the reported changes can be seen as different expressions of the same process; namely that the experience of anxiety and tension gives way to an increased basic trust and an increased reliance on one’s abilities. We fleshed out this idea in terms of changes in the participants’ fields of relevant affordances.

We then discussed the clinical implications of our findings. Participants stressed the importance of being properly informed beforehand on what to expect from treatment. Also, it would be worthwhile to engage partners and/or family-members in the treatment. Furthermore, many participants testify of the usefulness of including Cognitive Behavioural Therapy or a different form of psychotherapy within treatment. On the basis of our findings, we pointed to the limitations of current measures of treatment success and the current definition of treatment response. We ended by making several suggestions for further research.

Since DBS is a relatively new, experimental treatment for patients suffering from treatment-refractory Obsessive Compulsive Disorder (OCD) and other psychiatric disorders, much of its effects are still unknown. This makes it all the more important to carefully investigate patients’ own experiences in order to inform research as well as clinical practice.

## Supporting Information

S1 Table Overview of participants. (XLSX)

S1 Text Topiclist. (DOCX)

S2 Text Permission to publish Fig 1 under CC BY licence. (DOCX)
