# Supplementary material for: Effects of Deep Brain Stimulation on the Lived Experience of Obsessive-Compulsive Disorder Patients: In-Depth Interviews with 18 Patients
Source: PLoS One. 2015 Aug 27;10(8):e0135524. doi: 10.1371/journal.pone.0135524 (PMC4552296; doi:10.1371/journal.pone.0135524)
Supplement: S1 Text — (DOCX) [file pone.0135524.s002.docx]

Topiclist – Effects of DBS on the lived experience of OCD patients

I. Background

▪ Can you something about yourself and your background? And how you have ended up in this hospital?

▪ Can you tell me about your obsessions and/ or compulsions and how that works in your case?

II. Effects of Deep Brain Stimulation

*General*

▪ What are your experiences with DBS? What has changed following treatment with DBS?

*(1) Person*

Did you notice any changes after the DBS with regard to:

▪ your body, bodily sensations, bodily awareness, way of moving, or posture

▪ your perception (vision, taste, etc.)

▪ cognition: way of thinking, memory capacity, concentration, etc.

▪ mood and emotions

*(2) (Social) world*

▪ Has your world changed following DBS treatment?

▪ Has your interest in things changed? Have you started to get interested in different things? Or has the intensity of your interest changed?

▪ To what extent has something changes in your contact with other people? How do other people around you experience that?

*(3) Way of interacting*

Did you notice any changes after the DBS with regard to:

▪ attention (inward or outward directedness)

▪ way of acting (merging in actions, amount of conscious control, spontaneous actions)

*(4) Existential stance*

▪ Do you feel the treatment with DBS has changed you as a person? If yes, how do you feel about that? Do the people around you notice these changes? And if so, how do they evaluate these changes?

▪ Has anything changed with regard to your stance on your illness?

▪ To what extent has your experience of freedom changed as a result of DBS treatment?

▪ Has your experience of time changed following treatment?

III. Conclusion

▪ Are there any issues we did not talk about yet that are important for your experience of the DBS treatment?

▪ Do you have any suggestions for how DBS treatment could be improved?
