# Supplementary material for: Effects of Deep Brain Stimulation on the Lived Experience of Obsessive-Compulsive Disorder Patients: In-Depth Interviews with 18 Patients
Source: PLoS One. 2015 Aug 27;10(8):e0135524. doi: 10.1371/journal.pone.0135524 (PMC4552296; doi:10.1371/journal.pone.0135524)
Supplement: S2 Text — (DOCX) [file pone.0135524.s003.docx]

For reprinting (a modified version of) Figure 1 we have obtained written permission from the publisher of Frontiers in Human Neuroscience, where we first published the figure in a paper in 2013:

Dear Dr de Haan,

Thank you for your message. To reproduce one of your figures originally published in a Frontiers Journal, no permission is needed – the same applies should you wish to reproduce your article as you own the copyright of your article.

Nevertheless, the full citation of the original publication is mandatory. Please follow the following example: "Figure as originally published in Weiss SA and Faber DS (2010) Field effects in the CNS play functional roles Front. Neural Circuits 4:15. doi: 10.3389/fncir.2010.00015".

Please do not hesitate to contact me should you have any further questions.

Kind Regards

Robin Driscoll, PhD

Journal Operations Assistant

Frontiers | Human Neuroscience Editorial Office

Journal Manager: Lucia Brandi, PhD

EPFL Innovation Park - Building I

1015 Lausanne, Switzerland | T +41(0)21 510 17 11

www.frontiersin.org | twitter.com/FrontiersIn
